# A synthetic free fatty acid-regulated transgene switch in mammalian cells and mice

**DOI:** 10.1093/nar/gky805

**Published:** 2018-09-14

**Authors:** Ying Liu, Ghislaine Charpin-El Hamri, Haifeng Ye, Martin Fussenegger

**Affiliations:** 1 Department of Biosystems Science and Engineering, ETH Zurich, Mattenstrasse 26, CH-4058 Basel, Switzerland; 2 Département Génie Biologique, Université Claude Bernard 1, 43 Boulevard du 11 Novembre 1918, F-69100 Villeurbanne, France; 3 Shanghai Key Laboratory of Regulatory Biology, Institute of Biomedical Sciences and School of Life Sciences, East China Normal University, Dongchuan Road 500, Shanghai 200241, China; 4 Faculty of Science, University of Basel, Mattenstrasse 26, CH-4058 Basel, Switzerland

## Abstract

Trigger-inducible transgene expression systems are utilized in biopharmaceutical manufacturing and also to enable controlled release of therapeutic agents *in vivo*. We considered that free fatty acids (FFAs), which are dietary components, signaling molecules and important biomarkers, would be attractive candidates as triggers for novel transgene switches with many potential applications, e.g. in future gene- and cell-based therapies. To develop such a switch, we rewired the signal pathway of human G-protein coupled receptor 40 to a chimeric promoter triggering gene expression through an increase of intracellular calcium concentration. This synthetic gene switch is responsive to physiologically relevant FFA concentrations in different mammalian cell types grown in culture or in a bioreactor, or implanted into mice. Animal recipients of microencapsulated sensor cells containing this switch exhibited significant transgene induction following consumption of dietary fat (such as Swiss cheese) or under hyperlipidaemic conditions, including obesity, diabetes and lipodystrophy.

## INTRODUCTION

Synthetic trigger-controlled gene switches that enable adjustable and reversible fine-tuning of target protein production are required for functional genomic research (1), drug discovery (2), gene therapy (3), biopharmaceutical manufacturing (4) and tissue engineering (5). Already, huge advances in synthetic biology have led to the creation and engineering of artificial biological pathways, organisms and devices through reassembling catalogued and standardized biological units in a systematic, rational and predictable manner to obtain novel and useful functions (6), and a wide range of transgene control systems with unprecedented precision and reliability has been developed. These gene switches have become essential components for the construction of sophisticated transcription/translation networks such as oscillators (7), inter-cellular communication systems (8) and biocomputers (9). Since switches that use exogenous inducers may cause secondary side effects, there is a need for gene switches that are responsive to endogenous metabolites/biomarkers, and many such systems that interface directly with host metabolism/physiology have already been validated in animal models, including models of gout (10), hyperthyroidism (11), liver injury (12) and diabetes (13).

Free fatty acids (FFAs) are important energy sources for most body tissues and key components of cell membranes and various lipid classes (triglycerides, phospholipids, cholesteryl esters) (14). They also serve as second messengers regulating cellular processes (15), precursors to various lipid mediators (16), factors influencing protein acylation (17) and modulators of gene transcription (18) and signal transduction (19). All of these activities are largely dependent on the carbon-chain lengths of FFAs. Short-chain fatty acids have less than six carbons, medium-chain fatty acids have 6–12 carbons and long-chain fatty acids have >12 carbons (20). Medium- and long-chain FFAs are primary energy sources metabolized through β-oxidation in tissues and constitute the predominant types of fatty acids in the bloodstream (21). FFA levels are predominantly regulated via nutrition and lipolysis from triglycerides, cholesterol, lipoproteins and adipose tissues, depending upon the energy demands of the body (14). Therefore, circulating FFAs are an important and sensitive biomarker of physiological status. We thus considered that FFAs would be attractive candidates as triggers for novel transgene switches.

Human G-protein coupled receptor 40 (GPR40), which is abundantly expressed in the pancreas, functions as a receptor for medium- to long-chain FFAs (22,23). Here, capitalizing on its high sensitivity and broad range of activation by major serum FFAs at physiologically relevant concentrations (22–24), we adopted human GPR40 as a sensor module and rewired its signal activation to transgene expression through synthetic promoters in mammalian cells. We show that the optimized FFA-activated transgene switch (FATS) thus obtained can sense and report blood fatty acid levels induced by dietary fat consumption or chronically altered physiological states. We also show that it can provide dose- and time-specific control of product gene expression in a bioreactor.

## MATERIALS AND METHODS

### Design of plasmids

Comprehensive design and construction details for all expression vectors are provided in Table 1.

**Table 1.** Plasmids and oligonucleotides used and designed in this study

Plasmid	Description	Reference
pcDNA3.1(+)	Mammalian expression vector (P_hCMV_-MCS-pA).	Life Technologies
pSEAP2-control	Constitutive SEAP expression vector (P_SV40_-SEAP-pA).	Clontech
GPR40-FLAG	P_hCMV_-driven expression vector encoding human GPR40 tagged C-terminally with FLAG epitope (P_hCMV_-hGPR40-FLAG-pA).	(82)
pNifty-SEAP	NF-κB inducible SEAP expression vector (P_NF-κB-ELAM_-SEAP-pA).	InvivoGen
pHY30	P_NFAT_-inducible SEAP expression vector (P_NFAT_-SEAP-pA).	(83)
MKp37	P_hCMV_-driven expression vector encoding TetR-Elk-1 hybrid transcription factor (P_hCMV_-TetR-Elk-1-pA).	(84)
pMF111	TetR-responsive SEAP expression vector (*tet*O_7_-P_hCMVmin_-SEAP-pA).	(85)
pMSCV	Calcium responsive insulin expression vector (P_MSCV-CRE-SRE-NFAT_-insulin-pA).	(29)
pAT14	P_SRE-NFAT_-driven SEAP expression vector (P_SRE-NFAT_-SEAP-pA).	(86)
pKR135	Constitutive mammalian LSR expression vector (P_hCMV_-LSR-pA).	(63)
pMG10	Vector encoding a P_TtgR1_-driven SEAP expression unit (P_TtgR1-_SEAP-pA).	(63)
pYL1	P_CRE-SRE-NFAT_–driven SEAP expression vector (P_CRE-SRE-NFAT_-SEAP-pA). P_CRE-SRE-NFAT_ was PCR amplified from pMSCV using OYL11 (5′-GCCCCGCTCGAGCGCACCAGACAGTGACG-3′, *Xho*I underlined) and OYL12 (5′- CCCCCCAAGCTTCTGGAATTCGAGCTTCCATTAT-3′, *Hin*dIII underlined), digested with *Xho*I/*Hin*dIII and ligated into pSEAP2-control (*Xho*I/*Hin*dIII).	This work (GenBank accession no. MH594278)
pYL2	pUC57-derived vector containing 5 × AP-1 binding site, 5 × NF-κB binding site followed by an IFN-β promoter.	This work
pYL3	P_AP-1-NF-κB_–driven SEAP expression vector (P_AP-1-NF-κB_-SEAP-pA). P_AP-1-NF-κB_ was excised from pYL2 using *Xho*I/*Hin*dIII and cloned into pSEAP2-control (*Xho*I/*Hin*dIII).	This work
pYL4	P_hCMV_-driven hGPR40 expression vector (P_hCMV_-hGPR40-pA). hGPR40 was PCR amplified from GPR40-FLAG using OYL20 (5′- CCGCGGAAGCTTCGAATGAATTCGCCCACCATGGACCTGCCCCCGCAGCTCTCCTTC-3′ *Hin*dIII underlined) and OYL21 (5′- AGCTAGGTTAACTCTAGATTACTTCTGGGACTTGCCCCCTTGCGT-3′ *Xba*I underlined), digested with (*Hin*dIII/*Xba*I) and cloned into pcDNA3.1(+) (*Hin*dIII/*Xba*I).	This work (GenBank accession no. MH607407)
pYL5	P_CRE_-driven SEAP expression (P_CRE_-SEAP-pA). P_CRE_ was PCR amplified from pYL1 using OYL11 (5′-GCCCCGCTCGAGCGCACCAGACAGTGACG-3′, *Xho*I underlined) and OYL13 (5′- GGAGACAGATCTACCGGGGTTCTCCCAT-3′, *Bgl*II underlined), digested with (*Xho*I/*Bgl*II) and inserted into pYL1 (*Xho*I/*Bgl*II).	This work
pYL6	P_SRE_-driven SEAP expression (P_SRE_-SEAP-pA). P_SRE_ was PCR amplified from pYL1 using OYL14 (5′-CCGCCCCTCGAGCGGGAGGATGTCCATATTA-3′, *Xho*I underlined) and OYL15 (5′- CGAGGAAGATCTCGGGAGATGTCCTAATATGG-3′, *Bgl*II underlined), digested with (*Xho*I/*Bgl*II) and inserted into pYL1 (*Xho*I/*Bgl*II).	This work
pYL7	P_CRE-SRE_-driven SEAP expression (P_CRE-SRE_-SEAP-pA). P_CRE-SRE_ was PCR amplified from pYL1 using OYL11 (5′-GCCCCGCTCGAGCGCACCAGACAGTGACG-3′, *Xho*I underlined) and OYL12 (5′- CCCCCCAAGCTTCTGGAATTCGAGCTTCCATTAT-3′, *Hin*dIII underlined), digested with (*Xho*I/*Hin*dIII) and inserted into pYL1 (*Xho*I/*Hin*dIII).	This work

**Abbreviations: P_hCMV_**, human cytomegalovirus immediate early promoter; **P_SV40_**, simian virus 40 promoter; **P_hCMVmin_**, minimal version of P_hCMV_; **SEAP**, human placental secreted alkaline phosphatase; **pA**, polyadenylation signal; **ELAM**, endothelial cell-leukocyte adhesion molecule; **NF-κB**, nuclear factor kappa-light-chain-enhancer of activated B cells; **NFAT**, nuclear factor of activated T-cells; ***tet*O**, tetracycline-responsive operator; **TetR**, Tet repressor; **MSCV**, murine stem cell virus; **CRE**, cyclic adenosine monophosphate response element; **SRE**, serum response element; **AP-1**, activator protein 1; **IFN-β**, interferon beta.

### Cell culture and transfection

Human embryonic kidney cells (HEK-293T, ATCC: CRL-11268), baby hamster kidney cells (BHK-21, ATCC: CCL-10), human cervical adenocarcinoma cells (HeLa, ATCC: CCL-2), human fibrosarcoma cells (HT-1080, ATCC: CCL-121) and human bone marrow stromal cells immortalized with human telomerase reverse transcriptase (hMSC-hTERT) (25) were cultured in Dulbecco's modified Eagle's medium (DMEM, Invitrogen, Basel, Switzerland, cat. no. 52100–39) supplemented with 10% (v/v) fetal calf serum (FCS, Sigma-Aldrich, St. Louis, MO, USA, cat. no. F7524) and 1% (v/v) penicillin/streptomycin solution (Biowest, Nuaillé, France; cat. no. L0022–100). Wild-type Chinese hamster ovary cells (CHO-K1, ATCC: CCL-61) were grown in ChoMaster^®^ HTS (Cell Culture Technologies, Gravesano, Switzerland; cat. no. HTS-8) supplemented with 5% (v/v) FCS and 1% penicillin/streptomycin solution. FreeStyle^™^-293F suspension cells (Life Technologies, Carlsbad, CA; cat. no. R79007) were cultivated in FreeStyle^™^-293 Expression Medium (Life Technologies; cat. no. 12338018) supplemented with 1% penicillin/streptomycin solution and grown in flasks on an orbital shaker (IKA KS 260 basic; IKA-Werke GmbH, Staufen im Breisgau, Germany; cat. no. 0002980200) set to 100–150 rpm. All cell types were cultivated at 37°C in a humidified atmosphere containing 5% CO_2_. Cell number and viability were quantified using an electric field multichannel cell counting device (Casy Cell Counter and Analyzer Model TT, Roche Diagnostics GmbH). For transfection of BHK-21, CHO-K1, HeLa and HT-1080, 4 × 10^5^ cells seeded per well of a six-well plate 12 h before transfection were incubated overnight with a 4:1 PEI:DNA mixture solution (PEI: polyethyleneimine; MW 40 000, Polysciences, Inc., Warrington, USA; 1 mg/ml in water, pH 7.0), while a 3:1 PEI:DNA mixture was used for transfection of HEK-293T, hMSC-hTERT and FreeStyle^™^-293F cells. After transfection, cells were detached by incubation with 2 ml Trypsin–EDTA (Biowest, Nuaillé, France, cat. no. L0940) for 3 min at 37°C, collected in 8 ml cell culture medium, centrifuged for 3 min at 290 × g, and re-suspended with inducer-containing DMEM (3.5 ml/well of six-well plate) for re-seeding (300 μl/well for 48-well plate; 150 μl/well for 96-well plate). All cells were cultivated in their specific media containing different inducer concentrations, and reporter protein levels were assayed at the indicated times.

### SEAP quantification

The production of SEAP (human placental-secreted alkaline phosphatase) was quantified in cell culture supernatants as described previously (26). Serum levels of SEAP were profiled using a chemiluminescence-based assay (Roche Diagnostics GmbH, Mannheim, Germany).

### Inducer and reagents

GW9508 (cas. no. 885101-89-3, Sigma-Aldrich Chemie GmbH) was dissolved in DMSO to a final concentration of 25 mM as a stock solution. TAK875 (cas. no. A11018, AdooQ BioScience) was dissolved in DMSO to 12.5 mM. Palmitic acid (cas. no. 57103), *cis*-4,7,10,13,16,19-docosahexaenoic acid (cas. no. 6217545), linoleic acid (cas. no. 60333), oleic acid (cas. no. 112801), 5,8,11-eicosatriynoic acid (cas. no. 13488227), myristic acid (cas. no. 544638), stearic acid (cas. no. 57114), lauric acid (cas. no 143077), linolenic acid (cas. no. 463401), arachidonic acid (cas. no. 506321) and rosiglitazone (cas. no. 122320–73-4) were purchased from Sigma and diluted in DMSO to obtain 50 mM stock solutions. Butter, Swiss cheese, fresh whole milk, extra virgin olive oil, sesame oil and sunflower oil were purchased from Coop, Basel. Charcoal-stripped fetal bovine serum was from Invitrogen (cas. no. 12676-011).

### Animal experiments

Cell implants were produced by encapsulating pYL4/pYL1-transgenic HEK-293T cells into coherent alginate-poly-(l-lysine)-alginate beads (400 μm; 200 cells/capsule) using an Inotech Encapsulator Research Unit IE-50R (Buechi Labortechnik AG, Flawil, Switzerland) set to the following parameters: 25 mL syringe operated at a flow rate of 450 units, 200 μm nozzle with a vibration frequency of 1024 Hz and bead dispersion voltage of 1.2 kV, stirrer speed set at 4.5 units. Six-week-old male Swiss mice (oncins France souche 1, Charles River Laboratory, Lyon, France) weighing 25–28 g were intraperitoneally injected with 700 μl serum-free DMEM containing 2 × 10^6^ cells and were orally gavaged with oleic acid (300 μl; 44.5, 89 or 179 mg/mL) or Swiss cheese (300 μl, 1.00 g/ml in 4 M urea/1% SDS) and control solution (urea 4 M/1% SDS), twice daily. To prepare an HIV-associated lipodystrophy mouse model, wild-type male C57BL6 mice (The Jackson Laboratory, Maine, USA) at the age of 8 weeks were dosed via intragastric gavage twice daily with ritonavir (200 μl, 3 mg/ml) (Interchim, Code: R1519, France) or an equal volume of vehicle solution (7.8% Ethonal, 7.8% Tween 80 in water (v/v)) for 20 days. This dose corresponds to a clinically relevant concentration of the HIV protease inhibitor (30–60 mg/kg⋅d). Mice were fed on normal chow throughout the experiment and were fasted for 4–5 h before cell injection on the day of experiment. To prepare high fat (HF)-induced obese mice, 4-week-old male C57BL6 mice were kept in a temperature-controlled room (22 °C) on a 12-h light-dark cycle. Upon arrival, the mice were divided into two groups and fed on either a HF diet (60 kcal% fat diet, T-58Y1-58126, TestDiet, UK) or a control diet (4 kcal% fat diet; Teklad Global 2016 diet, Envigo, France) for 15 weeks. Body weight was measured weekly. As genetically disposed diabetic animals, *db/db* mice (female, 8 weeks old) were purchased from Janvier Labs. Blood samples were collected 24 h after treatment and serum was isolated using BD Microtainer^®^ SST tubes according to the manufacturer's instructions (centrifugation for 5 min at 10 000 g; Becton Dickinson, Plymouth, UK; cat. no. 365967). All experiments involving animals were performed according to the directives of the European Community Council (2010/63/EU), approved by the French Republic (project nos. DR2014-42 and DR2016-13), and carried out by Ghislaine Charpin-El Hamri (no. 69266309) and Marie Daoud-El Baba (no. 69266310) at the University of Lyon, Institut Universitaire de Technologie (IUT), F69622 Villeurbanne Cedex, France.

## RESULTS

### Design of an FFA-activated transgene switch (FATS) in mammalian cells

**Figure 1.**
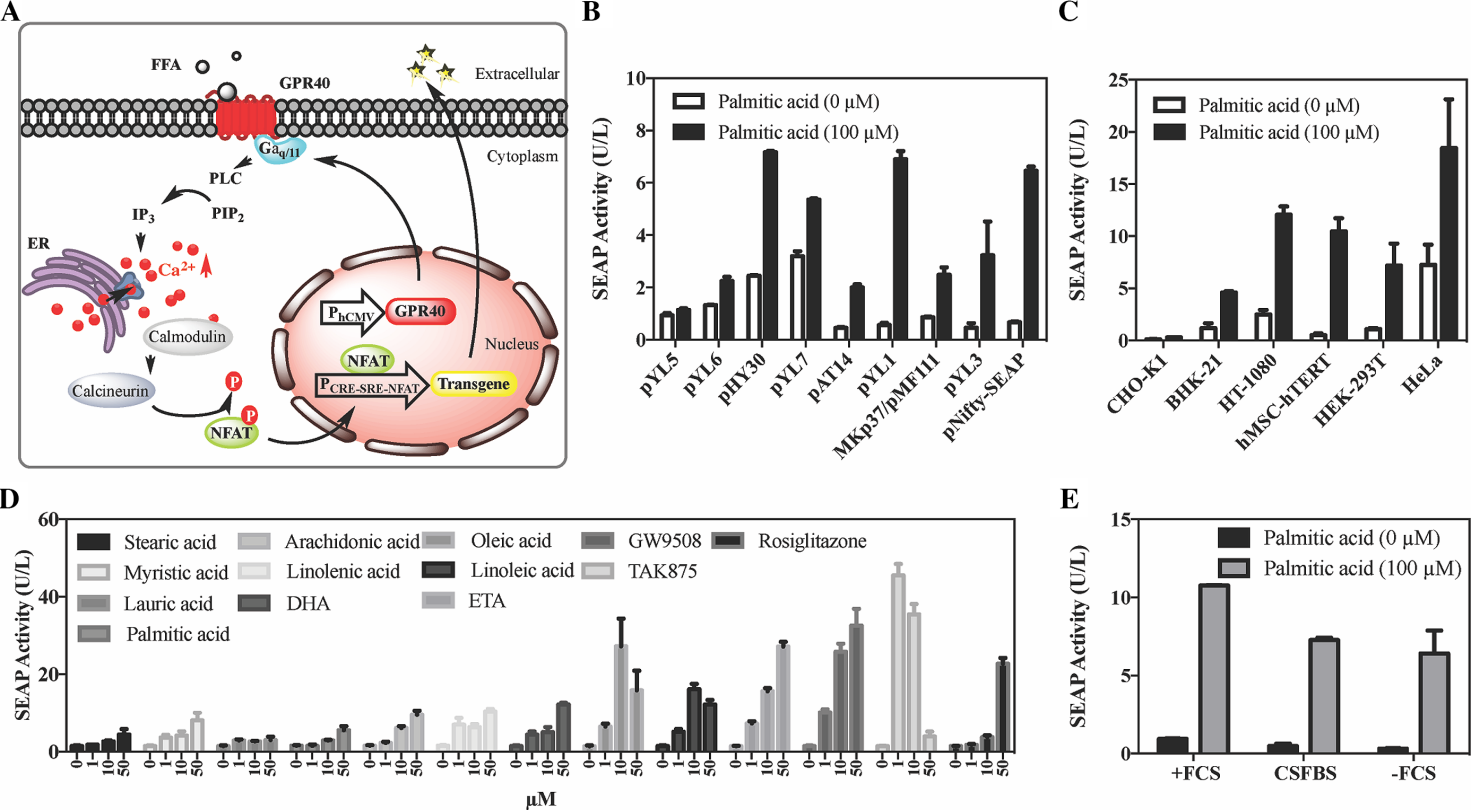
(**A**) Design of a transcription-control device activated by free fatty acids (FFAs). Interaction of human GPR40 with FFAs induces conformational changes that activate its coupled G protein subunit G_αq/11_, which in turn catalyzes PLC-mediated hydrolysis of phosphatidylinositol 4,5-bisphosphate (PIP_2_) to inositol triphosphate (IP_3_), leading to mobilization of endoplasmic reticulum (ER) Ca^2+^ stores. This cytoplasmic Ca^2+^ surge is sensed by calmodulin, which activates calcineurin, leading to the dephosphorylation of NFAT and translocation to the nucleus. Transgene expression is initiated through the binding of NFAT to a synthetic promoter (P_CRE-SRE-NFAT_) composed of three Ca^2+^ response elements in *cis* arrangement: CRE, SRE and NFAT. PLC, phospholipase C; P indicates a phosphate group; CRE, cyclic adenosine monophosphate response element; SRE, serum response element; NFAT, nuclear factor of activated T cell response element. (**B**) Optimization of FFA-controlled SEAP expression with different promoter configurations. HEK-293T cells were co-transfected with human GPR40-encoding expression vector (pYL4; P_hCMV_-hGPR40-pA) and an expression vector encoding SEAP under control of different promoters (pYL5, P_CRE_-SEAP-pA; pYL6, P_SRE_-SEAP-pA; pHY30, P_NFAT_-SEAP-pA; pYL7, P_CRE-SRE_-SEAP-pA; pAT14, P_SRE-NFAT_-SEAP-pA; pYL1, P_CRE-SRE-NFAT_-SEAP-pA; MKp37/pMF111, P_hCMV_-TetR-Elk-1-pA/*tet*O_7_-P_hCMVmin_-SEAP-pA; pYL3, P_AP-1-NF-κB_-SEAP-pA; pNifty-SEAP, P_NF-κB-ELAM_-SEAP-pA), and cultivated for 24 h in the presence of 100 μM palmitic acid. (**C**) Validation of FFA-inducible SEAP expression in different mammalian cells. Cells were transfected with pYL4 (P_hCMV_-hGPR40-pA)/pYL1 (pYL1, P_CRE-SRE-NFAT RE_-SEAP-pA) and incubated with 100 μM palmitic acid for 24 h. (**D**) Responsiveness of FATS to different fatty acids and synthetic molecules. pYL4/pYL1-transfected HEK-293T cells were incubated with various fatty acids and chemicals at increasing concentrations and SEAP expression was assayed after 24 h. DHA (*cis*-4,7,10,13,16,19-docosahexaenoic acid), ETA (5,8,11-eicosatriynoic acid). (**E**) Serum influence on system performance. Palmitic acid-inducible SEAP expression in pYL4/pYL1-transgenic HEK-293T cells cultivated in culture media containing 10% fetal calf serum (+FCS), 10% charcoal-stripped fetal bovine serum (CSFBS) or no serum (-FCS) after 24 h. All data are means ± SD (*n* = 3).

The constitutively expressed human GPR40 construct (pYL4, P_hCMV_-hGPR40-pA) senses extracellular FFA levels and triggers an increase of intracellular calcium through a Gα_q/11_-dependent signaling pathway (22,23). By rewiring the intracellular calcium surge via NFAT-dependent activation (27) of a calcium-responsive promoter (pYL1, P_CRE-SRE-NFAT_-SEAP-pA) containing cyclic adenosine monophosphate response elements (CRE), serum response element (SRE) and nuclear factor of activated T cell response element (NFAT) (29,30–32), extracellular FFA levels could be directly linked to the expression of a specific target gene (Figure 1A).

Next, we screened promoter variants with different calcium-responsive elements (31,33,34) in order to optimize the system. We constructed monomeric (P_CRE_, P_SRE_, P_NFAT_, P_NF-κB_), dimeric (P_CRE-SRE_, P_SRE-NFAT_, P_AP-1-NF-κB_) and trimeric (P_CRE-SRE-NFAT_) promoters, and observed the greatest transgene induction with P_CRE-SRE-NFAT_. The combinatorial assembly of these three response elements might enhance the sensitivity and amplitude of calcium regulation, and therefore, maximizes the overall signal transduction mechanism of the cell (Figure 1B).

Versatility of the optimized FFA-activated transgene switch (FATS) was assessed by co-transfection of pYL4 and pYL1 into several rodent and human cells. Consistent SEAP induction with palmitic acid indicated that the system was functional in all tested cell types, including stem cell-derived hMSC-hTERT, suggesting broad applicability of this gene control device (Figure 1C). Variations in GPR40 and related signaling protein expression (35), GPCR phosphorylation (36), cellular composition of downstream calcium signaling effectors and regulators (37,38), and protein secretion and transfection efficiencies (39) may possibly explain the different expression profiles in specific cell types and species of cell hosts. Considering the basal expression levels, maximum expression levels and induction fold, we selected two human-derived cell types, human embryonic kidney 293 cells (HEK-293T) and human bone marrow stromal cells transgenic for the catalytic subunit of human telomerase (hMSC-hTERT), for further characterization (Figure 1C).

Based on the broad sensitivity of human GPR40 to medium- and long-chain FFAs, we tested the system with a wide range of the most physiologically relevant FFAs and observed dose-dependent transcriptional activation in the concentration range from 1 to 50 μM (Figure 1D). Significant gene induction was also observed with synthetic GPR40 agonists proposed to have clinical potential for type-2 diabetes mellitus and hepatic steatosis, i.e. GW9508 (40), TAK-875 (fasiglifam) (41), and the anti-diabetic drug rosiglitazone (Avandia™) from the thiazolidinedione family (42) (Figure 1D). Thus, FATS may have potential applications in single drug-coordinated multiple therapeutics release, or in the combined therapy for a collective metabolic disorders.

The influence of fatty acids contained in the fetal calf serum (FCS) used to supplement standard cell culture media was assessed by cultivating pYL4/pYL1-cotransfected HEK-293T cells in medium containing no FCS or in medium containing charcoal-stripped fetal bovine serum, which is devoid of lipid-related components (Figure S1). This resulted in lower levels of basal and induced transgene expression, but had no major impact on the overall fold change of expression induced by the FATS system (Figure 1E).

### Characterization of FATS *in vitro*

**Figure 2.**
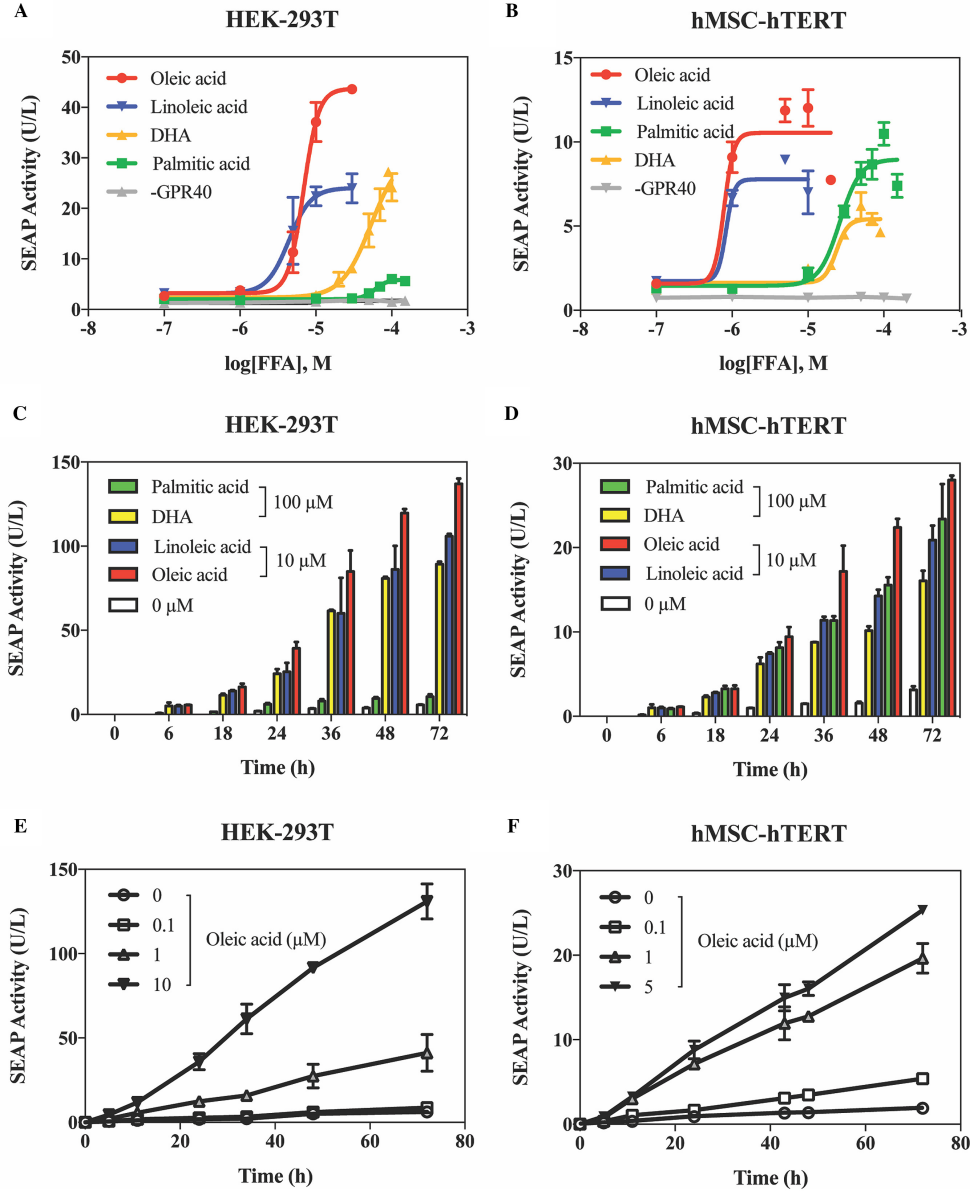
Responsiveness of FATS to individual fatty acids. Dose-responsive SEAP expression in pYL4/pYL1-transgenic (**A**) HEK-293T or (**B**) hMSC-hTERT cells co-cultured with specific fatty acids for 24 h. Control cells harboring only reporter (pYL1; P_CRE-SRE-NFAT_-SEAP-pA) were stimulated with palmitic acid. Induction kinetics of FATS with individual fatty acids in (**C**) HEK-293T or (**D**) hMSC-hTERT cells transfected with pYL4/pYL1 during cultivation for 72 h. Induction kinetics of FATS with increasing amounts of oleic acid in (**E**) HEK-293T or (**F**) hMSC-hTERT cells harboring pYL4/pYL1 during cultivation for 72 h. All data are means ± SD (*n* = 3).

Detailed characterization of the FATS system was done with oleic acid (OA, C18:1), palmitic acid (PA, C16:0), linoleic acid (LA, C18:2) and docosahexaenoic acid (DHA, C22:6) because these FFAs represent the major types of fatty acids that are present in human blood (20). When HEK-293T and hMSC-hTERT cells co-transfected with pYL4/pYL1 were treated with increasing concentrations of OA, LA, PA and DHA, concentration-dependent expression of SEAP was observed (Figure 2A, B). HEK-293T and hMSC-hTERT cells both showed the greatest sensitivity to OA and LA among the four FFAs. The activation was fully GPR40-dependent, as cells expressing reporter only (pYL1) showed no SEAP induction at any tested concentrations of PA (Figure 2A, B). These concentrations of fatty acids that are able to regulate the circuit had no impact on cell viability, since SEAP production by cells transfected with pSEAP2-control (P_SV40_-SEAP-pA) showed little or no dependence on FFAs at the testing concentrations (Figure S2); any negative impact of high concentrations of FFA on the metabolism or viability of the cells would be expected to impair their overall cellular transgene expression capacity.

**Figure 3.**
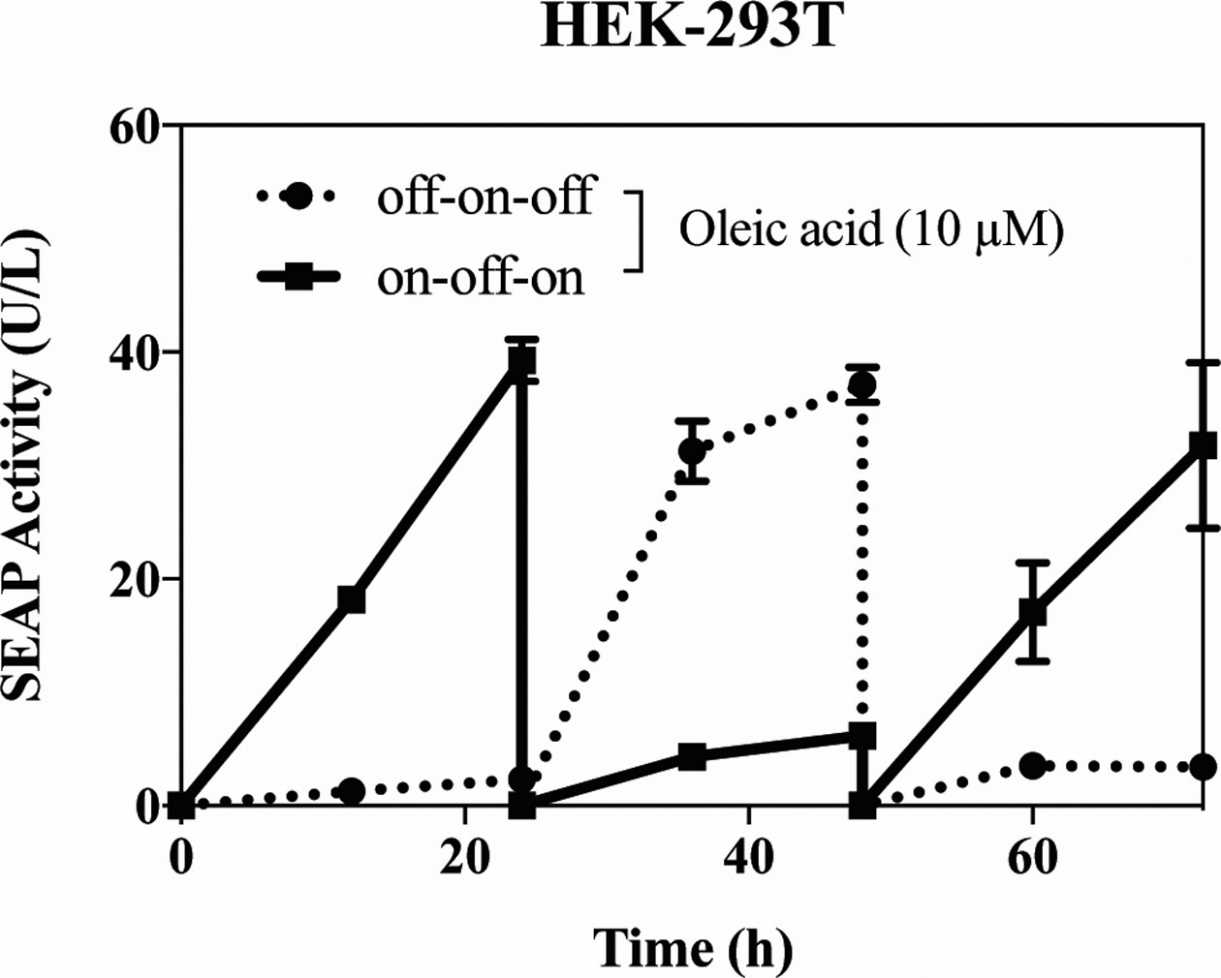
Reproducibility and reversibility of FATS-mediated gene expression in response to cycles of addition and removal of the inducer. pYL4/pYL1-transfected HEK-293T cells were cultured in the presence or absence of oleic acid. Every 24 h, the culture medium was exchanged and the cell density was re-adjusted. All data are means ± SD (*n* = 3).

When assayed at different time points and at increasing dosages of FFAs, FATS exhibited fast induction kinetics, affording a response within 6 h (Figure 2C, D) and a dose-dependent SEAP expression profile within 72 h (Figure 2E, F). The transgene switch also showed excellent reversibility in response to cycles of exposure to 0 and 10 μM OA at 24 h intervals (Figure 3).

**Figure 4.**
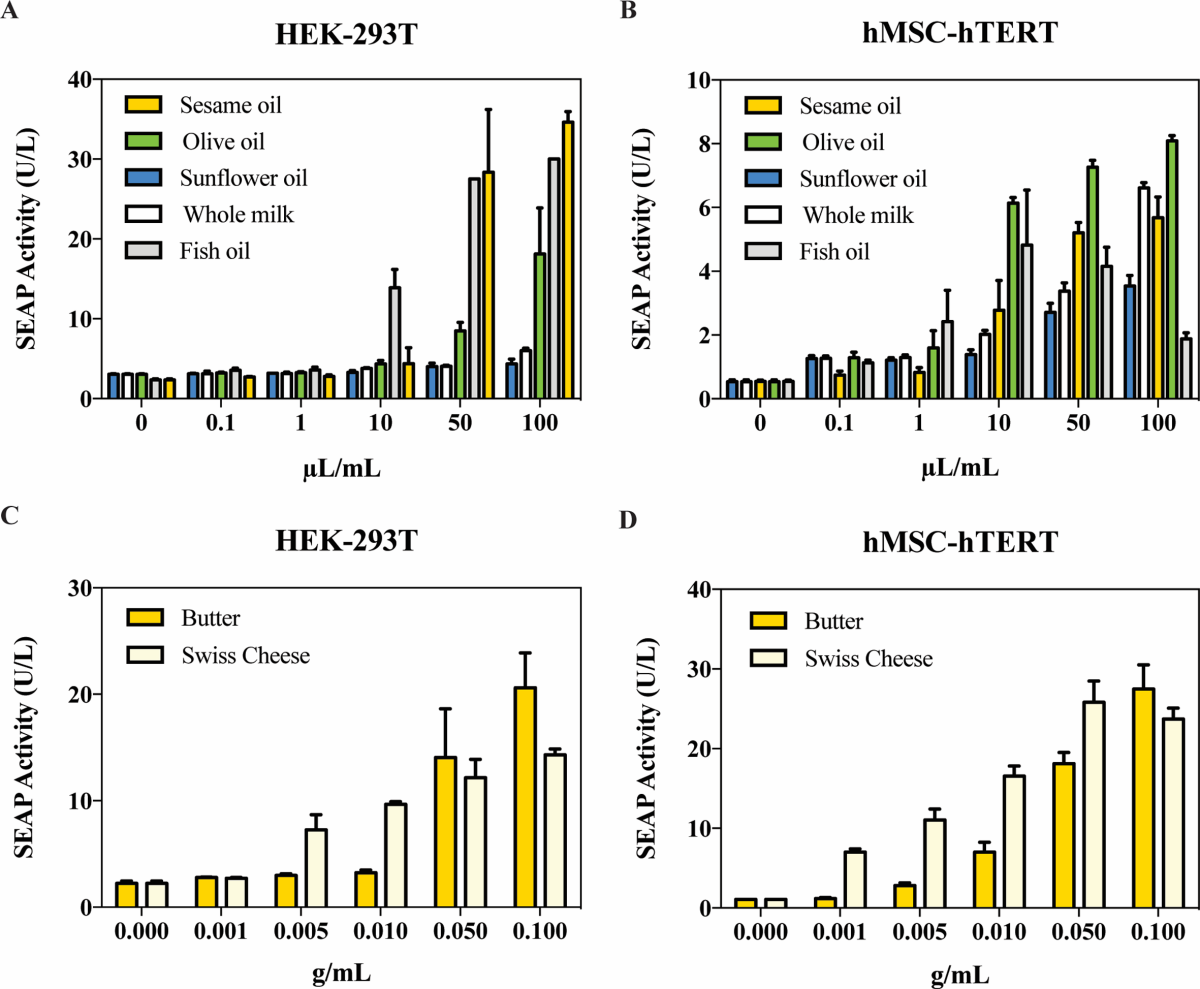
Dietary fat-induced SEAP expression. (**A**) HEK-293T and (**B**) hMSC-hTERT cells were transfected with pYL4/pYL1 and co-cultured with milk or oil at different concentrations for 24 h. pYL4/pYL1-transfected (**C**) HEK-293T and (**D**) hMSC-hTERT cells were cultivated in cell culture medium containing different concentrations of melted butter or cheese, and SEAP expression was assayed after 24 h. All data are means ± SD (*n* = 3).

In order to evaluate whether food-grade fat can also trigger the FATS system, we exposed pYL4/pYL1-transgenic HEK-293T and hMSC-hTERT cell cultures to different amounts of dietary fats, including vegetable oils, fish oil, milk, butter and cheese (Figure 4). The results indicated that the FATS system could be regulated by fatty foods *in vitro* in a dose-dependent manner (Figure 4).

### FFA-regulated protein production in bioprocessing

**Figure 5.**
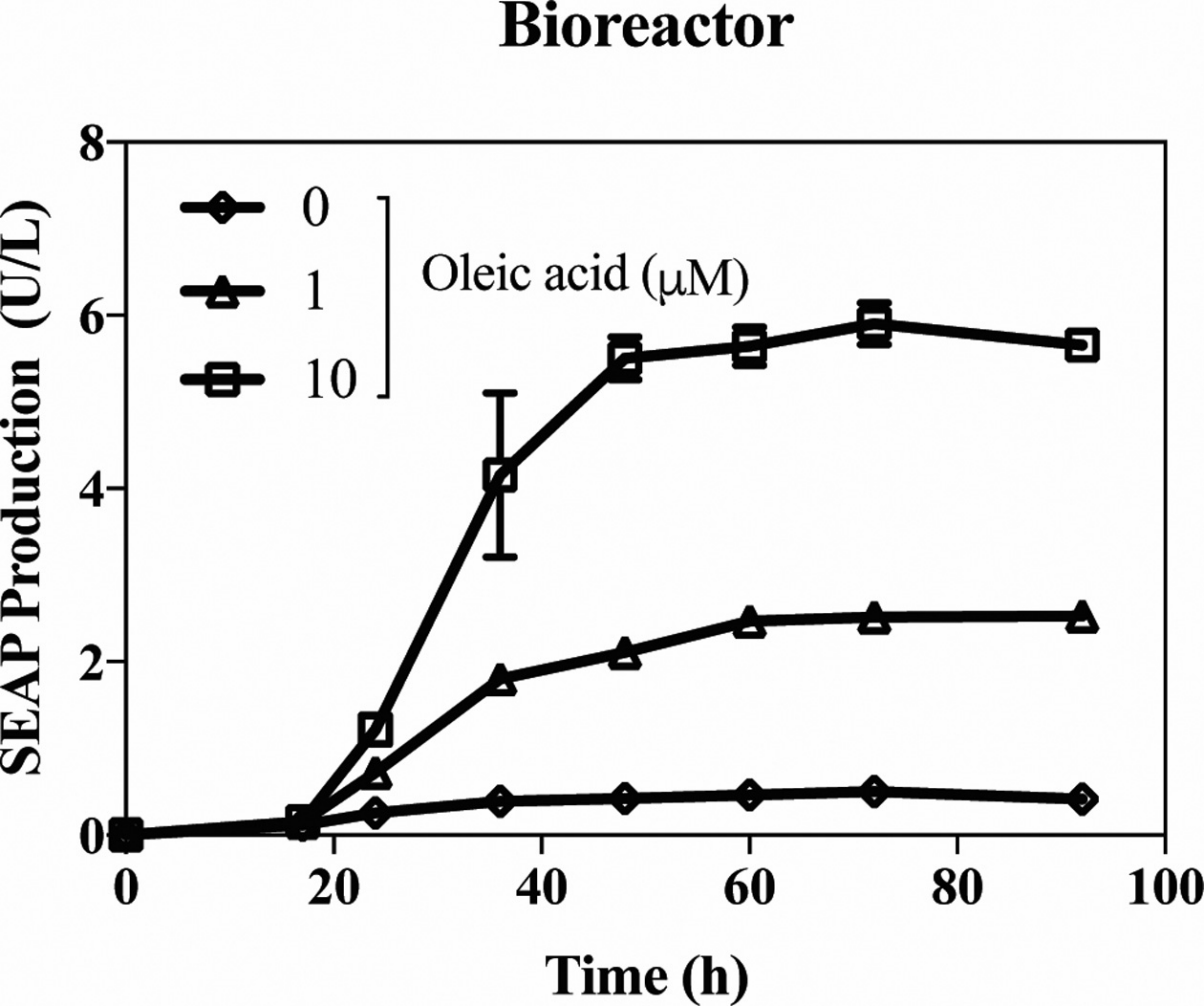
FFA-inducible product gene expression in bioreactors. SEAP production kinetics of pYL4/pYL1-transgenic Freestyle-293F suspension cells grown in bioreactors and triggered by the addition of 1 μM or 10 μM oleic acid for 92 h. Control bioreactors were run in the absence of oleic acid (0 μM). All data are means ± SD (*n* = 3).

Dose- and time-specific control of product gene expression in bioreactors requires the availability of gene switches responsive to trigger cues generally regarded as safe (GRAS) and licensed by the food and healthcare authorities. Fatty acids, as dietary components and cell metabolites, are therefore ideal trigger molecules in a biopharmaceutical manufacturing setting. We have tested oleic acid as a trigger compound for the timely induction of SEAP protein in HEK-293-derived serum-free suspension cultures, which are currently considered suitable for the production of viral particles for vaccines and gene therapy (43). In bioreactor operations, SEAP expression in pYL4/pYL1-transgenic HEK-293F cells was tightly repressed up to a specific concentration of added oleic acid, which could therefore be used to program the SEAP expression kinetics and the final titre of the gene product (Figure 5).

### Functional validation of FATS *in vivo*

**Figure 6.**
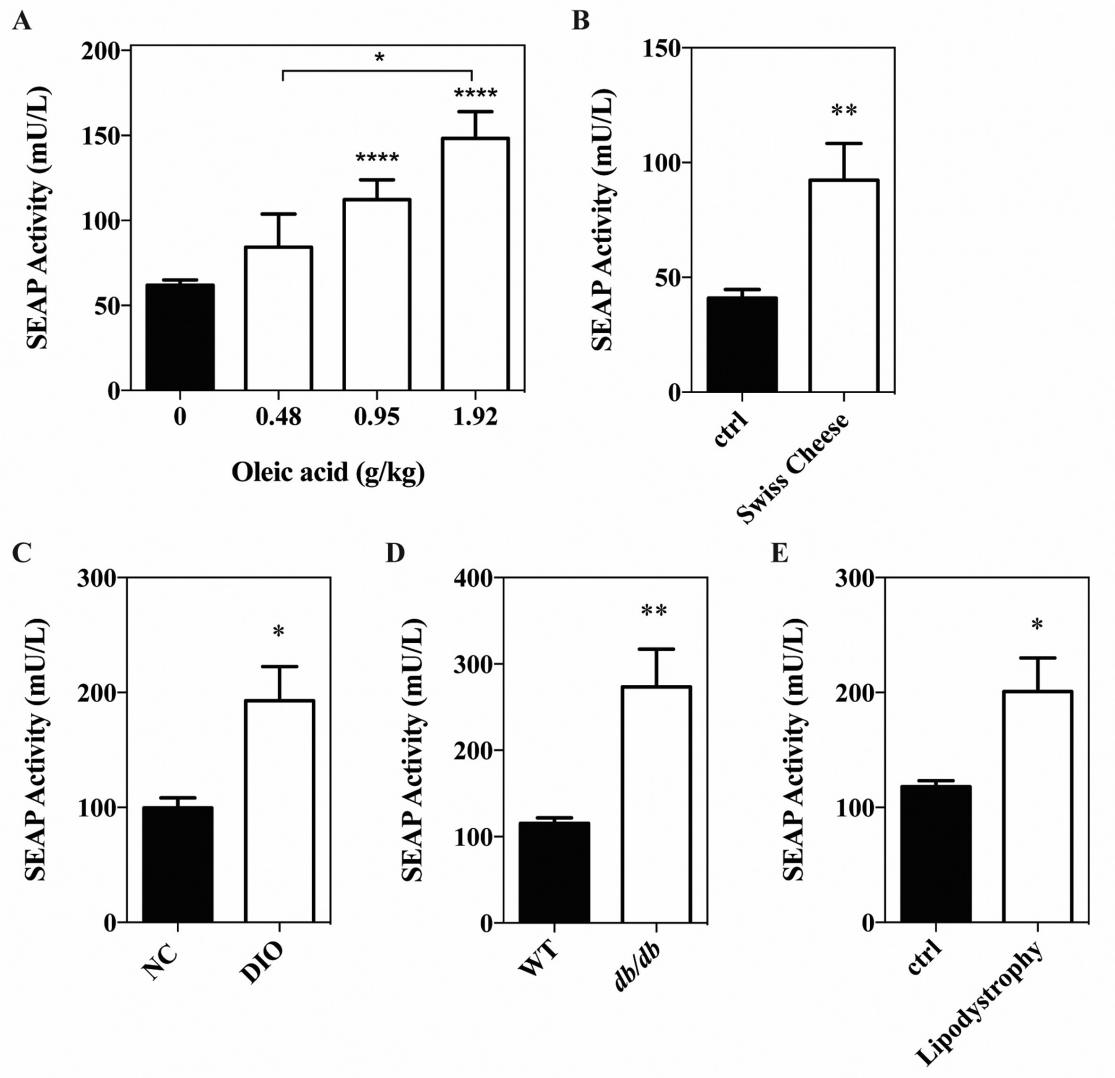
FATS-controlled transgene expression in mice. Mice implanted intraperitoneally with microencapsulated pYL4/pYL1-transgenic HEK-293T cells received (**A**) oleic acid (0 or 1.92 g/kg, *n* = 14; 0.48 or 0.95 g/kg, *n* = 7) or (**B**) Swiss cheese (10.7 g/kg, ***n*** = 7) by oral gavage twice daily, and blood SEAP activity was assayed after 24 h. (**C**) Mice were fed on HF diet (60 kcal% fat) (DIO) or normal chow (4 kcal% fat) (NC) for 15 weeks before receiving cell implants, and SEAP levels in the serum were assayed after 24 h (*n* = 7). (**D**) SEAP levels in diabetic *db/db* mice and their wild-type counterparts (WT) at 24 h after receiving cell implants (*n* = 7). (**E**) After receiving oral ritonavir (50 mg/kg⋅d) for 20 days, mice were injected with microencapsulated HEK_pYL4/pYL1_ cells and blood SEAP was assayed after 24 h (*n* = 7). Data are means ± SEM; statistics by two-tailed *t* test; ∗*P* < 0.05, ∗∗*P* < 0.01, ∗∗∗∗*P* < 0.0001 versus control or as indicated.

To validate the FATS system *in vivo*, we encapsulated pYL4/pYL1-transgenic HEK-293T cells in coherent alginate-poly-l-lysine-alginate microcapsules and implanted the microcapsules intraperitoneally into mice. The mice were given oleic acid at different doses (Figure 6A) or Swiss cheese (Figure 6B) by oral gavage twice daily. After 24 h, assay of blood samples showed dose-dependent and significant SEAP induction compared to controls. Next, to verify if the fat-sensor can be applied to detect pathological hyperlipidemia, we first tested the system on high fat (HF) diet-induced obesity (DIO) mice (DIO 42.79 ± 2.26 g versus ctrl 25.57 ± 0.67 g, *n* = 7, *P* < 0.0001). DIO mice bearing implant cells showed significant transgene expression after 24 h (Figure 6C). We then investigated the functionality of FATS in *db/db* mice lacking the leptin receptor, which develop type-2 diabetes (44) and show increased lipolysis of endogenous adipose stores, leading to increased levels of circulating FFAs (45). *db/db* mice implanted with microencapsulated cells showed significant transgene induction after 24 h (Figure 6D). Another common condition exhibiting high circulating fatty acids is lipodystrophy due to genetic issues, anti-retroviral medications or HIV infection (46). Long-term treatment of mice with ritonavir reproduces the clinical features of protease inhibitor-induced lipodystrophy in HIV-infected patients (47,48). Therefore, we tested the cell implants in a ritonavir–induced lipodystrophy mice model and observed significant system activation after 24 h (Figure 6E). In control mice implanted with cells constitutively expressing SEAP (pSEAP2-control), no significant difference in blood SEAP levels could be observed between non-treated animals and mice that either consumed fat or suffered from obesity (DIO), type-2 diabetes (db/db) and lipodystrophy (Figure S3). Furthermore, blood lipid levels were determined, and exhibited a good correlation with the SEAP expression profile *in vivo* (Table 2). Overall, the performance validation in mice represents a proof-of concept that the FATS system could be available as a gene switch to control desired transgene expression via intake of fatty foods. It may also be useful as a biosensor to monitor and correct pathological levels of fatty acids in a diagnostic or therapeutic setting.

**Table 2.** Blood fatty acid levels quantified after 24 h in different groups of mice

	Blood fatty acids (mmol/L)	
Mouse group	−	+
oleic acid	0.548 ± 0.031	0.681 ± 0.037 (0.48 g/kg)^a^
		0.765 ± 0.064 (0.95 g/kg)^b^
		0.945 ± 0.065 (1.92 g/kg)^c^
Swiss cheese	0.516 ± 0.061	0.714 ± 0.067^d^
DIO	0.717 ± 0.061	1.036 ± 0.097^e^
*db/db*	0.816 ± 0.045	1.373 ± 0.146^f^
Lipodystrophy	0.821 ± 0.050	0.980 ± 0.036^g^

^a^*P = 0.017 Mice receiving oleic acid (0.48 g/kg, *n* = 7; +) versus their vehicle control (*n* = 14; −).

^b^**P = 0.003 Mice receiving oleic acid (0.95 g/kg, *n* = 7; +) versus their vehicle control (*n* = 14; −).

^c^****P <0.0001 Mice receiving oleic acid (1.92 g/kg; +) versus their vehicle control (−) (*n* = 14).

^d^*P = 0.048 Mice receiving Swiss cheese (+) versus their vehicle control (−) (*n* = 7).

^e^*P = 0.016 Mice receiving high-fat diet (DIO) (+) versus mice fed on normal chow (NC) (−) (*n* = 7).

^f^**P = 0.003 *db/db* mice (+) versus their wild-type control (−) (*n* = 7).

^g^*P = 0.025 Lipodystrophy mice (receiving ritonavir) (+) versus their vehicle control (−) (*n* = 7).

Values are means ± SEM for each group.

## DISCUSSION

Gene switches reported so far have generally been based on prokaryotic repressors/activators together with targeted operators of the same origin (49), which raises concerns about possible pleiotropic effects in mammalian cells compared with regulatory systems derived from endogenous components (50). Indeed, adverse effects of increased immune responses to prokaryotes-derived transgene products (e.g. the widely used tetracycline-dependent systems) have been reported in many non-human primates, such as complete loss of transgene regulation and expression (51,52) and the development of anaphylactic reactions in humans, including cardiac arrest (53). Moreover, the triggering compounds of conventional gene switches, such as antibiotics (28,54), immunosuppressive agents (55), drugs (56) or hormones and their derivatives (57,58), may have secondary therapeutic effects and collateral side effects (59) including physiological disruption of the host and development of antibiotic resistance (60). There are also economic considerations associated with the elimination of inducer molecules during downstream purification of biopharmaceuticals (61). All of these issues have greatly hindered the widespread implementation of gene switches in biopharmaceutical manufacturing and biomedical applications (62). On the other hand, by targeting on endogenous metabolites as regulatory compounds and employing exclusively human-derived cellular components as building blocks, FATS can directly communicate with patients’ metabolic networks to interface with and respond to endogenous signals, and so offers better biocompatibility than previous hybrid systems (63). The transmembrane localization and broad sensing capacity of GPR40 also equip the system with higher sensitivity (down to 0.1 μM) than previously reported nuclear-receptor based system (63) (Figure S4).

The functionality of the FATS device using a safe trigger molecule to achieve timely remote control of product gene expression in a proof-of-concept bioprocessing manufacturing setting suggests that FATS has great potential for application in manufacturing drug and tool proteins (64) without introducing major issues regarding downstream purification, validation, or approval.

Furthermore, the versatile adaptability of FATS to different mouse models indicates that it is a promising candidate for future diagnostic and therapeutic applications. Free fatty acid levels in the blood are normally regulated to within a certain range in healthy individuals. However, a pathological increase can be maintained for a long time without causing any significant signs or symptoms, while current clinical discrete measurements are far from ideal either for early diagnosis or for guiding preventive measures (65,66).

Specifically, dietary interventions through food monitoring are often unsuccessful due to the hidden nature of many fats, the variation of type and content of fatty acids contained in foods, and the sensitivity of individuals to questions about fat intake in their diets (67). For these reasons, a fat-sensor to reflect endogenous fatty acid levels through a readily accessible reporter molecule would be particularly desirable in real clinical settings. This is important, because elevated blood concentrations of fatty acids increase oxidant stress, promote endothelial dysfunction, induce inflammatory cytokine release and provoke insulin resistance (68). High blood FFA levels are seen in obesity (69), insulin resistance (70), type 2 diabetes mellitus (71), cardiovascular disease (72) and hepatic steatosis (73), and moreover, interventions to decrease circulating fatty acid levels have shown great therapeutic value in improving insulin sensitivity, normalizing glucose homeostasis in type-2 diabetes mellitus and correcting dyslipidemia in cardiovascular complications (74). When linked to the production of a hypolipidemic agent (75–77), the fat-sensor could be customized to enable versatile therapeutic interventions and self-autonomously restore blood lipid homeostasis in populations highly susceptible to hyperlipidaemia and its complications. For example, the present findings in lipodystrophic mice suggest the feasibility of an alternative FATS-controlled cell-based therapy for lipoatrophic diabetes via combined expression of leptin and adiponectin, which has been shown to completely reverse insulin resistance in lipoatrophic mice (78). Furthermore, by changing the output protein to ghrelin (79) or progestins (80), the circuit might be applied to prevent cancer cachexia patients from experiencing significant whole-body lipolysis in the late stage of chemotherapy (81). Thus, we believe the FATS system, combining the use of a physiologically compatible inducer, precise and reversible transcription tunability, and broad functionality in different cell types or in bioreactors, as well as in animals, has a wide range of potential applications in advanced protein manufacturing, diagnosis of pathological blood-fat levels as well as gene- and cell-based therapies.

## Supplementary Material

Supplementary Data

## References

[1] Malleret G., Haditsch U., Genoux D., Jones M.W., Bliss T.V., Vanhoose A.M., Weitlauf C., Kandel E.R., Winder D.G., Mansuy I.M. Inducible and reversible enhancement of learning, memory, and long-term potentiation by genetic inhibition of calcineurin. Cell. 2001; 104:675–686. 11257222 10.1016/s0092-8674(01)00264-1

[2] Weber W., Fussenegger M. The impact of synthetic biology on drug discovery. Drug Discov. Today. 2009; 14:956–963. 19580884 10.1016/j.drudis.2009.06.010 PMC7108258

[3] Naldini L. Gene therapy returns to centre stage. Nature. 2015; 526:351–360. 26469046 10.1038/nature15818

[4] Weber W., Schoenmakers R., Keller B., Gitzinger M., Grau T., Daoud-El Baba M., Sander P., Fussenegger M. A synthetic mammalian gene circuit reveals antituberculosis compounds. PNAS. 2008; 105:9994–9998. 18621677 10.1073/pnas.0800663105 PMC2481315

[5] Ehrbar M., Schoenmakers R., Christen E.H., Fussenegger M., Weber W. Drug-sensing hydrogels for the inducible release of biopharmaceuticals. Nat. Mater. 2008; 7:800–804. 18690239 10.1038/nmat2250

[6] Xie M., Fussenegger M. Designing cell function: assembly of synthetic gene circuits for cell biology applications. Nat. Rev. Mol. Cell Biol. 2018; 19:507–525. 29858606 10.1038/s41580-018-0024-z

[7] Tigges M., Marquez-Lago T.T., Stelling J., Fussenegger M. A tunable synthetic mammalian oscillator. Nature. 2009; 457:309–312. 19148099 10.1038/nature07616

[8] Bacchus W., Fussenegger M. Engineering of synthetic intercellular communication systems. Metab. Eng. 2013; 16:33–41. 23246522 10.1016/j.ymben.2012.12.001

[9] Daniel R., Rubens J.R., Sarpeshkar R., Lu T.K. Synthetic analog computation in living cells. Nature. 2013; 497:619–623. 23676681 10.1038/nature12148

[10] Kemmer C., Gitzinger M., Daoud-El Baba M., Djonov V., Stelling J., Fussenegger M. Self-sufficient control of urate homeostasis in mice by a synthetic circuit. Nat. Biotechnol. 2010; 28:355–360. 20351688 10.1038/nbt.1617

[11] Saxena P., Charpin-El Hamri G., Folcher M., Zulewski H., Fussenegger M. Synthetic gene network restoring endogenous pituitary-thyroid feedback control in experimental Graves' disease. PNAS. 2016; 113:1244–1249. 26787873 10.1073/pnas.1514383113 PMC4747754

[12] Bai P., Ye H.F., Xie M.Q., Saxena P., Zulewski H., Charpin-El Hamri G., Djonov V., Fussenegger M. A synthetic biology-based device prevents liver injury in mice. J. Hepatol. 2016; 65:84–94. 27067456 10.1016/j.jhep.2016.03.020 PMC4914822

[13] Xie M., Ye H., Wang H., Charpin-El Hamri G., Lormeau C., Saxena P., Stelling J., Fussenegger M. beta-cell-mimetic designer cells provide closed-loop glycemic control. Science. 2016; 354:1296–1301. 27940875 10.1126/science.aaf4006

[14] Coppack S.W., Jensen M.D., Miles J.M. In vivo regulation of lipolysis in humans. J. Lipid Res. 1994; 35:177–193. 8169522

[15] Nishizuka Y. Membrane phospholipid degradation and protein kinase C for cell signalling. Neurosci. Res. 1992; 15:3–5. 1336582 10.1016/0168-0102(92)90013-3

[16] Smith W.L. Nutritionally essential fatty acids and biologically indispensable cyclooxygenases. Trends Biochem. Sci. 2008; 33:27–37. 18155912 10.1016/j.tibs.2007.09.013

[17] Resh M.D. Fatty acylation of proteins: the long and the short of it. Prog Lipid Res. 2016; 63:120–131. 27233110 10.1016/j.plipres.2016.05.002 PMC4975971

[18] Chapkin R.S., McMurray D.N., Davidson L.A., Patil B.S., Fan Y.Y., Lupton J.R. Bioactive dietary long-chain fatty acids: emerging mechanisms of action. Br. J. Nutr. 2008; 100:1152–1157. 18492298 10.1017/S0007114508992576 PMC2648819

[19] Pepino M.Y., Kuda O., Samovski D., Abumrad N.A. Structure-function of CD36 and importance of fatty acid signal transduction in fat metabolism. Annu. Rev. Nutr. 2014; 34:281–303. 24850384 10.1146/annurev-nutr-071812-161220 PMC4329921

[20] Tvrzicka E., Kremmyda L.-S., Stankova B., Zak A. Fatty acids as biocompounds: their role in human metabolism, health and disease – a review. part 1: classification, dietary sources and biological functions. Biomed. Pap. 2011; 155:117–130. 10.5507/bp.2011.038 21804620

[21] Goodman D.S., Shiratori T. Fatty acid composition of human plasma lipoprotein fractions. J. Lipid Res. 1964; 5:307–313. 5873366

[22] Itoh Y., Kawamata Y., Harada M., Kobayashi M., Fujii R., Fukusumi S., Ogi K., Hosoya M., Tanaka Y., Uejima H. Free fatty acids regulate insulin secretion from pancreatic beta cells through GPR40. Nature. 2003; 422:173–176. 12629551 10.1038/nature01478

[23] Briscoe C.P., Tadayyon M., Andrews J.L., Benson W.G., Chambers J.K., Eilert M.M., Ellis C., Elshourbagy N.A., Goetz A.S., Minnick D.T. The orphan G protein-coupled receptor GPR40 is activated by medium and long chain fatty acids. J. Biol. Chem. 2003; 278:11303–11311. 12496284 10.1074/jbc.M211495200

[24] Burtis C.A., Ashwood E.R., Border B., Tietz N.W. Tietz Fundamentals of Clinical Chemistry. 2001; 5th edn Philadelphia: W.B. Saunders.

[25] Simonsen J.L., Rosada C., Serakinci N., Justesen J., Stenderup K., Rattan S.I., Jensen T.G., Kassem M. Telomerase expression extends the proliferative life-span and maintains the osteogenic potential of human bone marrow stromal cells. Nat. Biotechnol. 2002; 20:592–596. 12042863 10.1038/nbt0602-592

[26] Schlatter S., Rimann M., Kelm J., Fussenegger M. SAMY, a novel mammalian reporter gene derived from Bacillus stearothermophilus alpha-amylase. Gene. 2002; 282:19–31. 11814674 10.1016/s0378-1119(01)00824-1

[27] Hogan P.G., Chen L., Nardone J., Rao A. Transcriptional regulation by calcium, calcineurin, and NFAT. Genes Dev. 2003; 17:2205–2232. 12975316 10.1101/gad.1102703

[28] Fussenegger M., Morris R.P., Fux C., Rimann M., von Stockar B., Thompson C.J., Bailey J.E. Streptogramin-based gene regulation systems for mammalian cells. Nat. Biotechnol. 2000; 18:1203–1208. 11062442 10.1038/81208

[29] Stanley S.A., Gagner J.E., Damanpour S., Yoshida M., Dordick J.S., Friedman J.M. Radio-Wave heating of iron oxide nanoparticles can regulate plasma glucose in mice. Science. 2012; 336:604–608. 22556257 10.1126/science.1216753 PMC3646550

[30] Bading H. Nuclear calcium signalling in the regulation of brain function. Nat. Rev. Neurosci. 2013; 14:593–608. 23942469 10.1038/nrn3531

[31] Hardingham G.E., Bading H. Calcium as a versatile second messenger in the control of gene expression. Microsc. Res. Tech. 1999; 46:348–355. 10504212 10.1002/(SICI)1097-0029(19990915)46:6<348::AID-JEMT3>3.0.CO;2-A

[32] Crabtree G.R., Schreiber S.L. SnapShot: Ca2+-calcineurin-NFAT signaling. Cell. 2009; 138:210. 19596245 10.1016/j.cell.2009.06.026 PMC2958059

[33] Ye R.D. Regulation of nuclear factor kappaB activation by G-protein-coupled receptors. J. Leukoc. Biol. 2001; 70:839–848. 11739545

[34] Macian F., Lopez-Rodriguez C., Rao A. Partners in transcription: NFAT and AP-1. Oncogene. 2001; 20:2476–2489. 11402342 10.1038/sj.onc.1204386

[35] Atwood B.K., Lopez J., Wager-Miller J., Mackie K., Straiker A. Expression of G protein-coupled receptors and related proteins in HEK293, AtT20, BV2, and N18 cell lines as revealed by microarray analysis. BMC Genomics. 2011; 12:14. 21214938 10.1186/1471-2164-12-14 PMC3024950

[36] Tobin A.B., Butcher A.J., Kong K.C. Location, location, location … site-specific GPCR phosphorylation offers a mechanism for cell-type-specific signalling. Trends Pharmacol. Sci. 2008; 29:413–420. 18606460 10.1016/j.tips.2008.05.006 PMC2880250

[37] Preuss A.K., Connor J.A., Vogel H. Transient transfection induces different intracellular calcium signaling in CHO K1 versus HEK 293 cells. Cytotechnology. 2000; 33:139–145. 19002821 10.1023/A:1008150402616 PMC3466731

[38] Berridge M.J., Lipp P., Bootman M.D. The versatility and universality of calcium signalling. Nat. Rev. Mol. Cell Biol. 2000; 1:11–21. 11413485 10.1038/35036035

[39] Dalton A.C., Barton W.A. Over-expression of secreted proteins from mammalian cell lines. Protein Sci. 2014; 23:517–525. 24510886 10.1002/pro.2439 PMC4005704

[40] Li M., Meng X., Xu J., Huang X., Li H., Li G., Wang S., Man Y., Tang W., Li J. GPR40 agonist ameliorates liver X receptor-induced lipid accumulation in liver by activating AMPK pathway. Sci. Rep. 2016; 6:25237. 27121981 10.1038/srep25237 PMC4848522

[41] Kaku K., Enya K., Nakaya R., Ohira T., Matsuno R. Efficacy and safety of fasiglifam (TAK-875), a G protein-coupled receptor 40 agonist, in Japanese patients with type 2 diabetes inadequately controlled by diet and exercise: a randomized, double-blind, placebo-controlled, phase III trial. Diabetes Obesity Metab. 2015; 17:675–681. 10.1111/dom.12467 PMC4676912 25787200

[42] Kersten S., Desvergne B., Wahli W. Roles of PPARs in health and disease. Nature. 2000; 405:421–424. 10839530 10.1038/35013000

[43] Dumont J., Euwart D., Mei B., Estes S., Kshirsagar R. Human cell lines for biopharmaceutical manufacturing: history, status, and future perspectives. Crit Rev. Biotechnol. 2016; 36:1110–1122. 26383226 10.3109/07388551.2015.1084266 PMC5152558

[44] Aasum E., Hafstad A.D., Severson D.L., Larsen T.S. Age-dependent changes in metabolism, contractile function, and ischemic sensitivity in hearts from db/db mice. Diabetes. 2003; 52:434–441. 12540618 10.2337/diabetes.52.2.434

[45] Murray A.J., Panagia M., Hauton D., Gibbons G.F., Clarke K. Plasma free fatty acids and peroxisome proliferator-activated receptor alpha in the control of myocardial uncoupling protein levels. Diabetes. 2005; 54:3496–3502. 16306367 10.2337/diabetes.54.12.3496

[46] Caron-Debarle M., Lagathu C., Boccara F., Vigouroux C., Capeau J. HIV-associated lipodystrophy: from fat injury to premature aging. Trends Mol. Med. 2010; 16:218–229. 20400373 10.1016/j.molmed.2010.03.002

[47] Periard D., Telenti A., Sudre P., Cheseaux J.J., Halfon P., Reymond M.J., Marcovina S.M., Glauser M.P., Nicod P., Darioli R. Atherogenic dyslipidemia in HIV-infected individuals treated with protease inhibitors. The Swiss HIV Cohort Study. Circulation. 1999; 100:700–705. 10449690 10.1161/01.cir.100.7.700

[48] Xu A., Yin S., Wong L., Chan K.W., Lam K.S. Adiponectin ameliorates dyslipidemia induced by the human immunodeficiency virus protease inhibitor ritonavir in mice. Endocrinology. 2004; 145:487–494. 14592951 10.1210/en.2003-1140

[49] Weber W., Fussenegger M. Molecular diversity-the toolbox for synthetic gene switches and networks. Curr. Opin. Chem. Biol. 2011; 15:414–420. 21470897 10.1016/j.cbpa.2011.03.003

[50] Goverdhana S., Puntel M., Xiong W., Zirger J.M., Barcia C., Curtin J.F., Soffer E.B., Mondkar S., King G.D., Hu J. Regulatable gene expression systems for gene therapy applications: progress and future challenges. Mol. Ther. 2005; 12:189–211. 15946903 10.1016/j.ymthe.2005.03.022 PMC2676204

[51] Le Guiner C., Stieger K., Snyder R.O., Rolling F., Moullier P. Immune responses to gene product of inducible promoters. Curr. Gene Ther. 2007; 7:334–346. 17979680 10.2174/156652307782151461

[52] Lanitis E., Poussin M., Hagemann I.S., Coukos G., Sandaltzopoulos R., Scholler N., Powell D.J. Redirected antitumor activity of primary human lymphocytes transduced with a fully human Anti-mesothelin chimeric receptor. Mol. Ther. 2012; 20:633–643. 22127019 10.1038/mt.2011.256 PMC3293635

[53] Maus M.V., Haas A.R., Beatty G.L., Albelda S.M., Levine B.L., Liu X.J., Zhao Y.B., Kalos M., June C.H. T cells expressing chimeric antigen receptors can cause anaphylaxis in humans. Cancer Immunol. Res. 2013; 1:26–31. 10.1158/2326-6066.CIR-13-0006 PMC3888798 24777247

[54] Weber W., Fux C., Daoud-el Baba M., Keller B., Weber C.C., Kramer B.P., Heinzen C., Aubel D., Bailey J.E., Fussenegger M. Macrolide-based transgene control in mammalian cells and mice. Nat. Biotechnol. 2002; 20:901–907. 12205509 10.1038/nbt731

[55] Rollins C.T., Rivera V.M., Woolfson D.N., Keenan T., Hatada M., Adams S.E., Andrade L.J., Yaeger D., van Schravendijk M.R., Holt D.A. A ligand-reversible dimerization system for controlling protein-protein interactions. PNAS. 2000; 97:7096–7101. 10852943 10.1073/pnas.100101997 PMC16505

[56] Tascou S., Sorensen T.K., Glenat V., Wang M.P., Lakich M.M., Darteil R., Vigne E., Thuillier V. Stringent rosiglitazone-dependent gene switch in muscle cells without effect on myogenic differentiation. Mol. Ther. 2004; 9:637–649. 15120324 10.1016/j.ymthe.2004.02.013

[57] Nordstrom J.L. Antiprogestin-controllable transgene regulation in vivo. Curr. Opin. Biotechnol. 2002; 13:453–458. 12459337 10.1016/s0958-1669(02)00356-7

[58] Palli S.R., Kapitskaya M.Z., Potter D.W. The influence of heterodimer partner ultraspiracle/retinoid X receptor on the function of ecdysone receptor. FEBS J. 2005; 272:5979–5990. 16302963 10.1111/j.1742-4658.2005.05003.x

[59] Lautermann J., Dehne N., Schacht J., Jahnke K. Aminoglycoside- and cisplatin-ototoxicity: From basic science to clinics. Laryngo Rhino Otol. 2004; 83:317–323. 10.1055/s-2004-814280 15143449

[60] Woolhouse M., Waugh C., Perry M.R., Nair H. Global disease burden due to antibiotic resistance - state of the evidence. J. Glob. Health. 2016; 6:010306. 27350872 10.7189/jogh.06.010306 PMC4920009

[61] Palomares L.A., Estrada-Mondaca S., Ramirez O.T. Production of recombinant proteins: challenges and solutions. Methods Mol. Biol. 2004; 267:15–52. 15269414 10.1385/1-59259-774-2:015

[62] Weber W., Fussenegger M. Emerging biomedical applications of synthetic biology. Nat. Rev. Genet. 2012; 13:21–35. 10.1038/nrg3094 PMC7097403 22124480

[63] Rossger K., Charpin-El-Hamri G., Fussenegger M. A closed-loop synthetic gene circuit for the treatment of diet-induced obesity in mice. Nat. Commun. 2013; 4:2825. 24281397 10.1038/ncomms3825 PMC3868331

[64] Weber W., Fussenegger M. Inducible product gene expression technology tailored to bioprocess engineering. Curr. Opin. Biotechnol. 2007; 18:399–410. 17933507 10.1016/j.copbio.2007.09.002

[65] Pilz S., Marz W. Free fatty acids as a cardiovascular risk factor. Clin. Chem. Lab. Med. 2008; 46:429–434. 18605928 10.1515/CCLM.2008.118

[66] Boden G. Obesity, insulin resistance and free fatty acids. Curr. Opin. Endocrinol. Diabetes Obes. 2011; 18:139–143. 21297467 10.1097/MED.0b013e3283444b09 PMC3169796

[67] Arab L. Biomarkers of fat and fatty acid intake. J. Nutr. 2003; 133:925s–932s. 12612178 10.1093/jn/133.3.925S

[68] Plutzky J. Expansion and contraction: the mighty, mighty fatty acid. Nat. Med. 2009; 15:618–619. 19498375 10.1038/nm0609-618 PMC8493568

[69] Koutsari C., Jensen M.D. Thematic review series: patient-oriented research. Free fatty acid metabolism in human obesity. J. Lipid Res. 2006; 47:1643–1650. 16685078 10.1194/jlr.R600011-JLR200

[70] Frohnert B.I., Jacobs D.R., Steinberger J., Moran A., Steffen L.M., Sinaiko A.R. Relation between serum free fatty acids and adiposity, insulin resistance, and cardiovascular risk factors from adolescence to adulthood. Diabetes. 2013; 62:3163–3169. 23670973 10.2337/db12-1122 PMC3749355

[71] Grapov D., Adams S.H., Pedersen T.L., Garvey W.T., Newman J.W. Type 2 diabetes associated changes in the plasma non-esterified fatty acids, oxylipins and endocannabinoids. PLoS One. 2012; 7:e48852. 23144998 10.1371/journal.pone.0048852 PMC3493609

[72] Pilz S., Scharnagl H., Tiran B., Wellnitz B., Seelhorst U., Boehm B.O., Marz W. Elevated plasma free fatty acids predict sudden cardiac death: a 6.85-year follow-up of 3315 patients after coronary angiography. Eur. Heart J. 2007; 28:2763–2769. 17766282 10.1093/eurheartj/ehm343

[73] Zhang J.W., Zhao Y., Xu C.F., Hong Y.N., Lu H.L., Wu J.P., Chen Y. Association between serum free fatty acid levels and nonalcoholic fatty liver disease: a cross-sectional study. Sci. Rep. 2014; 4:5832. 25060337 10.1038/srep05832 PMC5376058

[74] Eleftheriadou I., Grigoropoulou P., Katsilambros N., Tentolouris N. The effects of medications used for the management of diabetes and obesity on postprandial lipid metabolism. Curr. Diabetes Rev. 2008; 4:340–356. 18991602 10.2174/157339908786241133

[75] Yamauchi T., Kamon J., Waki H., Imai Y., Shimozawa N., Hioki K., Uchida S., Ito Y., Takakuwa K., Matsui J. Globular adiponectin protected ob/ob mice from diabetes and ApoE-deficient mice from atherosclerosis. J. Biol. Chem. 2003; 278:2461–2468. 12431986 10.1074/jbc.M209033200

[76] Xu A., Wang Y., Keshaw H., Xu L.Y., Lam K.S., Cooper G.J. The fat-derived hormone adiponectin alleviates alcoholic and nonalcoholic fatty liver diseases in mice. J. Clin. Invest. 2003; 112:91–100. 12840063 10.1172/JCI17797 PMC162288

[77] Tschop M.H., Finan B., Clemmensen C., Gelfanov V., Perez-Tilve D., Muller T.D., DiMarchi R.D. Unimolecular polypharmacy for treatment of diabetes and obesity. Cell Metab. 2016; 24:51–62. 27411008 10.1016/j.cmet.2016.06.021

[78] Yamauchi T., Kamon J., Waki H., Terauchi Y., Kubota N., Hara K., Mori Y., Ide T., Murakami K., Tsuboyama-Kasaoka N. The fat-derived hormone adiponectin reverses insulin resistance associated with both lipoatrophy and obesity. Nat. Med. 2001; 7:941–946. 11479627 10.1038/90984

[79] Strasser F., Lutz T.A., Maeder M.T., Thuerlimann B., Bueche D., Tschop M., Kaufmann K., Holst B., Brandle M., von Moos R. Safety, tolerability and pharmacokinetics of intravenous ghrelin for cancer-related anorexia/cachexia: a randomised, placebo-controlled, double-blind, double-crossover study. Br. J. Cancer. 2008; 98:300–308. 18182992 10.1038/sj.bjc.6604148 PMC2361459

[80] Maltoni M., Nanni O., Scarpi E., Rossi D., Serra P., Amadori D. High-dose progestins for the treatment of cancer anorexia-cachexia syndrome: a systematic review of randomised clinical trials. Ann. Oncol. 2001; 12:289–300. 11332139 10.1023/a:1011156811739

[81] Agustsson T., Wikrantz P., Ryden M., Brismar T., Isaksson B. Adipose tissue volume is decreased in recently diagnosed cancer patients with cachexia. Nutrition. 2012; 28:851–855. 22480800 10.1016/j.nut.2011.11.026

[82] Stoddart L.A., Brown A.J., Milligan G. Uncovering the pharmacology of the G protein-coupled receptor GPR40: high apparent constitutive activity in guanosine 5′-O-(3-[35S]thio)triphosphate binding studies reflects binding of an endogenous agonist. Mol. Pharmacol. 2007; 71:994–1005. 17200419 10.1124/mol.106.031534

[83] Ye H., Daoud-El Baba M., Peng R.W., Fussenegger M. A synthetic optogenetic transcription device enhances blood-glucose homeostasis in mice. Science. 2011; 332:1565–1568. 21700876 10.1126/science.1203535

[84] Keeley M., Busch J., Singh R., Abel T. TetR hybrid transcription factors report cell signaling and are inhibited by doxycycline. BioTechniques. 2005; 39:529–536. 16235565 10.2144/000112002

[85] Fussenegger M., Morris R.P., Fux C., Rimann M., von Stockar B., Thompson C.J., Bailey J.E. Streptogramin-based gene regulation systems for mammalian cells. Nat. Biotechnol. 2000; 18:1203–1208. 11062442 10.1038/81208

[86] Tastanova A., Folcher M., Muller M., Camenisch G., Ponti A., Horn T., Tikhomirova M.S., Fussenegger M. Synthetic biology-based cellular biomedical tattoo for detection of hypercalcemia associated with cancer. Sci. Transl. Med. 2018; 10:eaap8562. 29669854 10.1126/scitranslmed.aap8562

