# Supplementary material for: A synthetic free fatty acid-regulated transgene switch in mammalian cells and mice
Source: Nucleic Acids Res. 2018 Sep 14;46(18):9864–74. doi: 10.1093/nar/gky805 (PMC6182168; doi:10.1093/nar/gky805)
Supplement: Supplementary Data [file gky805_supplemental_files.docx]

**Supplementary Information**

**“A Synthetic Free Fatty Acid-Regulated Transgene Switch in Mammalian Cells and Mice”**

Ying Liu, Ghislaine Charpin-El Hamri, Haifeng Ye, Martin Fussenegger^*^

*To whom correspondence should be addressed. E-mail: fussenegger@bsse.ethz.ch

**This file includes:**

Supplementary Figures S1 to S4

**Supplementary Figures**

**
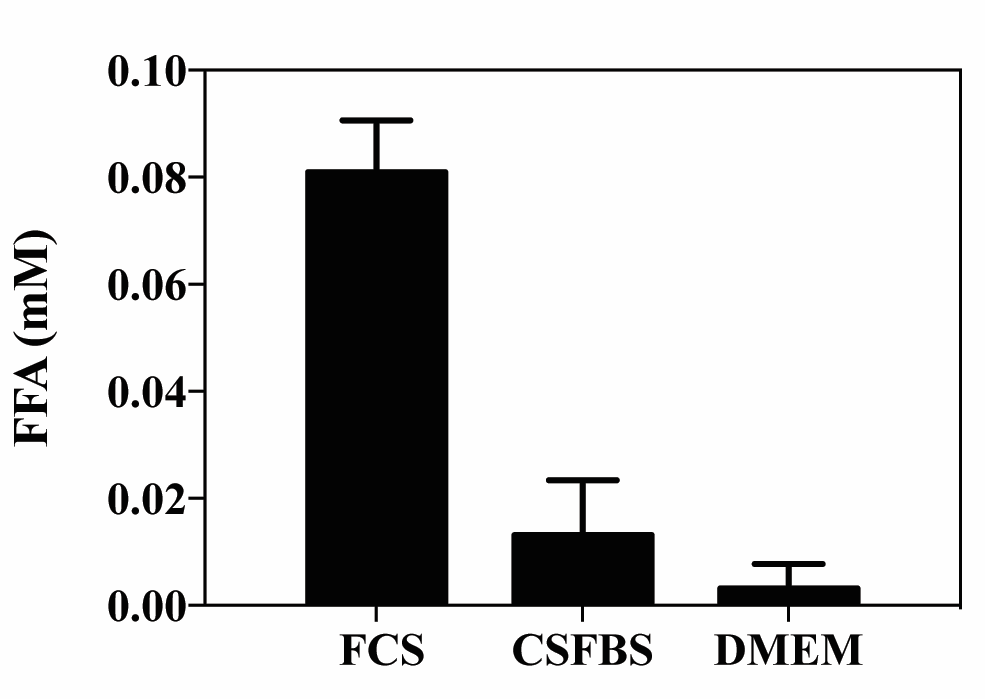
**

**Figure S1**. Fatty acids levels in fetal calf serum (FCS), charcoal-stripped fetal bovine serum (FBS) and pure cell culture medium (DMEM). All data are means ± SD (n = 3).


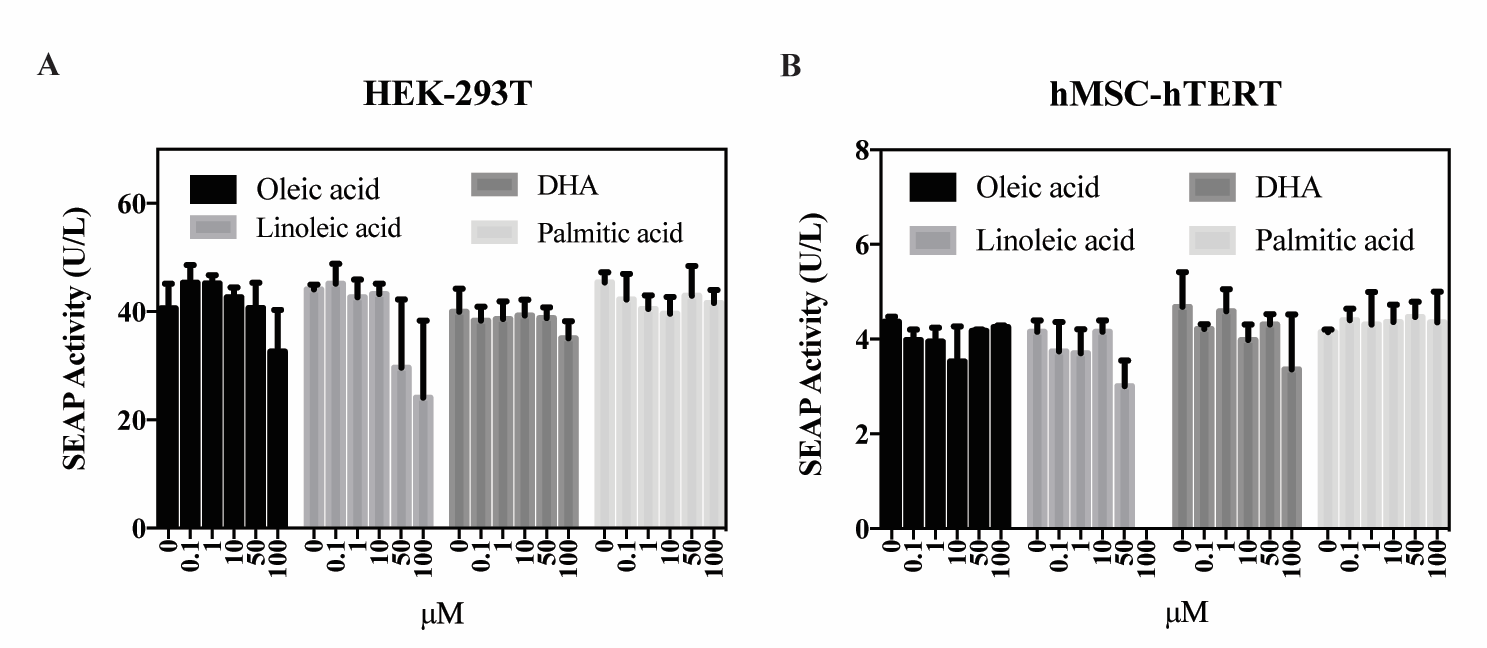


**Figure S2**. Toxicity of different fatty acids to mammalian cells. Impact of various concentrations of fatty acids on SEAP production by **(A)** HEK-293T cells and **(B)** hMSC-hTERT cells transfected with pSEAP2-control (P_SV40_-SEAP-pA) after 24 h. All data are means ± SD (n = 3).

**
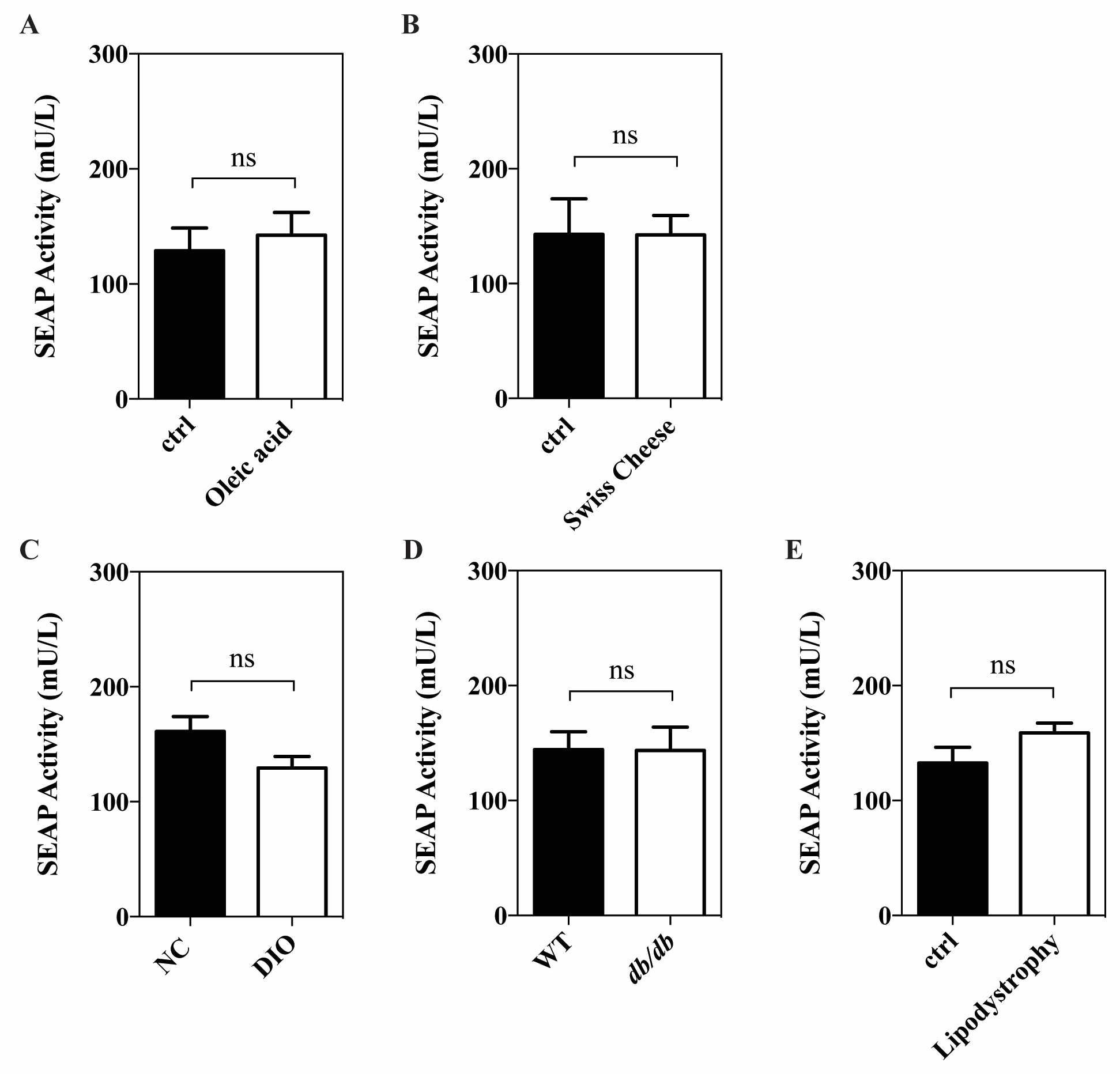
**

**Figure S3**. Control experiments in mice. HEK-293T cells transfected with pSEAP2-control were microencapsulated and implanted intraperitoneally. Blood SEAP activity was assayed after 24 h in all groups. **(A)** Oleic acid (1.9 g/kg), or **(B)** Swiss cheese (10.7 g/kg) was administered to mice by oral gavage twice daily (n = 7). **(C)** Mice were fed on HF diet (60 kcal% fat) (DIO) or normal chow (4 kcal% fat) (NC) for 31 weeks before receiving cell implants (n = 10 mice). **(D)** Diabetic *db/db* mice and their wild-type counterparts (WT) (n = 7 mice). **(E)** Mice were given oral ritonavir (50 mg/kg⋅d) for 14 days before receiving implantation of microencapsulated cells (ctrl n = 6; Lipodystrophy n = 5 mice). Data are means ± SEM; statistics by two-tailed *t* test; ns, not significant.


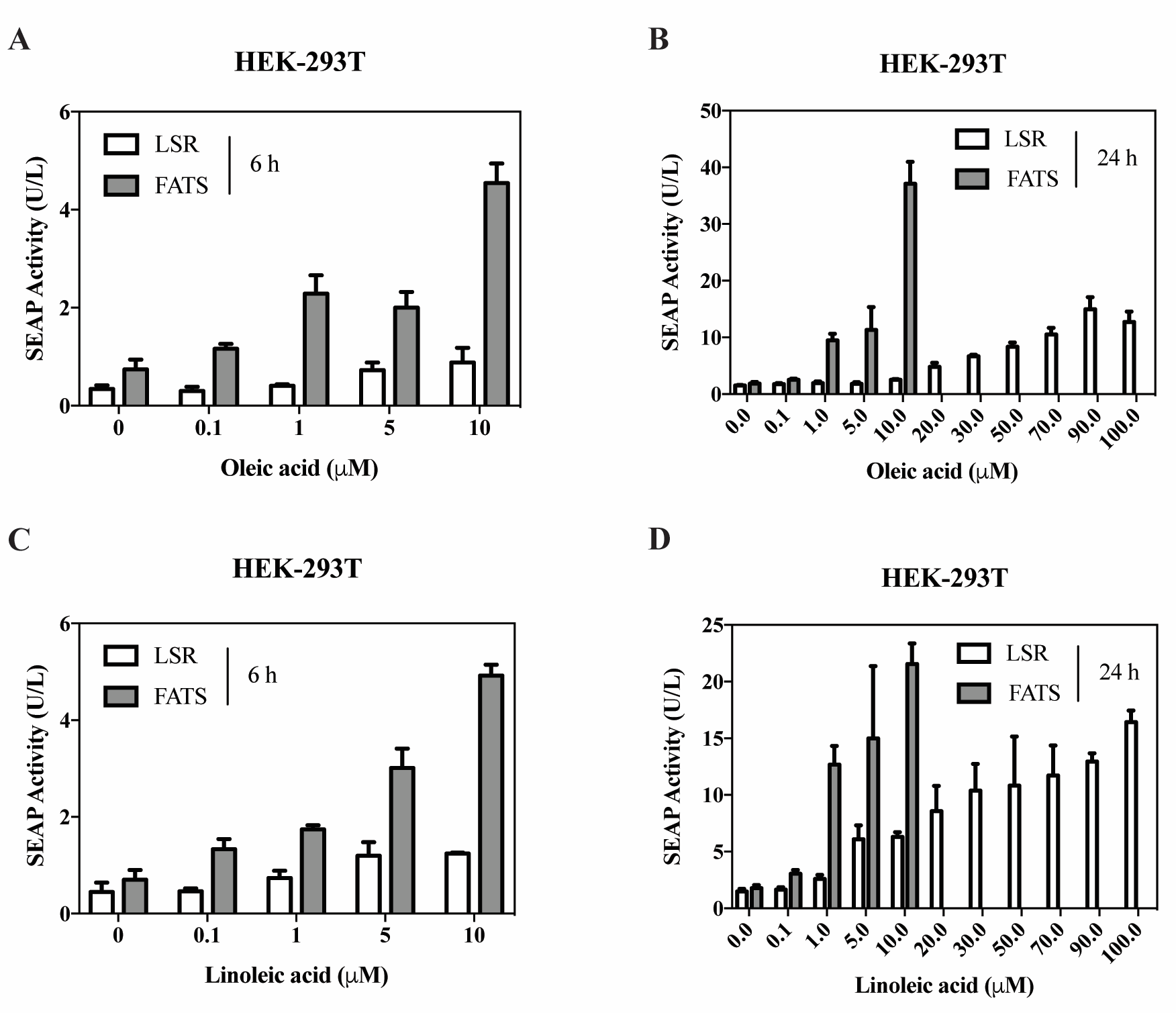


**Figure S4**. Comparative performance analysis of FATS and LRS gene switches. HEK-293T cells were transfected with either LSR (pKR135, P_hCMV_-LSR-pA /pMG10, P_TtgR1-_SEAP-pA) or FATS (pYL4 /pYL1) genetic components, and cultivated in cell culture medium containing different amounts of fatty acids. SEAP activity was assayed at indicated time point. Dose-dependent SEAP induction by oleic acid at different concentrations after **(A)** 6 h and **(B)** 24 h. Dose-dependent linoleic acid-inducible SEAP expression at different concentrations after **(C)** 6 h and **(D)** 24 h. All data are means ± SD (n = 3).
